# Identification of mycoparasitism-related genes against the phytopathogen *Sclerotinia sclerotiorum* through transcriptome and expression profile analysis in *Trichoderma harzianum*

**DOI:** 10.1186/1471-2164-15-204

**Published:** 2014-03-18

**Authors:** Andrei Stecca Steindorff, Marcelo Henrique Soller Ramada, Alexandre Siqueira Guedes Coelho, Robert Neil Gerard Miller, Georgios Joannis Pappas, Cirano José Ulhoa, Eliane Ferreira Noronha

**Affiliations:** 1 Departamento de Biologia Celular, Universidade de Brasília, Campus Universitário Darcy Ribeiro, Instituto de Ciências Biológicas, CEP 70.910-900 Brasília, DF, Brazil; 2 EMBRAPA Recursos Genéticos e Biotecnologia, Parque Estação Biológica, CP 02372 CEP 70.770-900 Brasília, DF, Brazil; 3 Escola de Agronomia e Engenharia de Alimentos, Universidade Federal de Goiás, Campus Samambaia, P.O. Box 131CEP 74001-970 Goiânia, GO, Brasil; 4 Departamento de Bioquímica e Biologia Molecular, Universidade Federal de Goiás, Campus Samambaia, Instituto de Ciências Biológicas, CEP 74.090-900 Goiânia, GO, Brazil

**Keywords:** *T. harzianum*, *S. sclerotiorum*, RNA-seq, Gene expression, Mycoparasitism

## Abstract

**Background:**

The species of *T. harzianum* are well known for their biocontrol activity against plant pathogens. However, few studies have been conducted to further our understanding of its role as a biological control agent against *S. sclerotiorum*, a pathogen involved in several crop diseases around the world. In this study, we have used RNA-seq and quantitative real-time PCR (RT-qPCR) techniques in order to explore changes in *T. harzianum* gene expression during growth on cell wall of *S. sclerotiorum* (SSCW) or glucose. RT-qPCR was also used to examine genes potentially involved in biocontrol, during confrontation between *T. harzianum* and *S. sclerotiorum*.

**Results:**

Data obtained from six RNA-seq libraries were aligned onto the *T. harzianum* CBS 226.95 reference genome and compared after annotation using the Blast2GO suite. A total of 297 differentially expressed genes were found in mycelia grown for 12, 24 and 36 h under the two different conditions: supplemented with glucose or SSCW. Functional annotation of these genes identified diverse biological processes and molecular functions required during *T. harzianum* growth on SSCW or glucose. We identified various genes of biotechnological value encoding proteins with functions such as transporters, hydrolytic activity, adherence, appressorium development and pathogenesis. To validate the expression profile, RT-qPCR was performed using 20 randomly chosen genes. RT-qPCR expression profiles were in complete agreement with the RNA-Seq data for 17 of the genes evaluated. The other three showed differences at one or two growth times. During the confrontation assay, some genes were up-regulated during and after contact, as shown in the presence of SSCW which is commonly used as a model to mimic this interaction.

**Conclusions:**

The present study is the first initiative to use RNA-seq for identification of differentially expressed genes in *T. harzianum* strain TR274, in response to the phytopathogenic fungus *S. sclerotiorum*. It provides insights into the mechanisms of gene expression involved in mycoparasitism of *T. harzianum* against *S.sclerotiorum.* The RNA-seq data presented will facilitate improvement of the annotation of gene models in the draft *T. harzianum* genome and provide important information regarding the transcriptome during this interaction.

## Background

*Sclerotinia sclerotiorum* (Lib.) de Bary is one of the most devastating and cosmopolitan plant pathogens. This fungus infects over 400 species of plants worldwide including important crops and numerous weeds [1]. *S. sclerotiorum* poses a threat to crops such as sunflower, soybean, oilseed rape, edible dry bean, chickpea, peanut, dry pea, lentils, onion and tulip [1]. It is capable of infecting flowers, leaves, fruits or stems [2] and its life cycle initiates by the germination of sclerotia and formation of infectious propagules. During the crop growing season, which dependson a set of environmental factors, fungal sclerotia germinate to form mycelia, which can directly infect host plants, or produce ascospores. Further, ascospores develop forming apothecia [3]. Ascospores are the primary infective propagules of *S. sclerotiorum* on many crops, and also can act in disease scattering since they may be transported to neighboring fields as well as over longer distances [1].

Chemical treatment is today the main strategy employed worldwide for fungal disease control. In order to develop alternative and sustainable methods for control of white mold, which do not cause negative environmental or economic impacts during crop production, as observed with the routine use of fungicides, our research group has isolated strains of a number of *Trichoderma* species from diverse agro-ecosystems in Brazil and assessed their potential for biocontrol of *S. sclerotiorum*. This analysis has included evaluation of antagonistic capacity, production of cell wall-degrading enzymes and production of volatile antibiotics [4]. Our results have identified *T. harzianum* Rifai (anamorph) strain TR274 as a promising biocontrol agent against *S.sclerotiorum in vitro* and under field conditions [5,6].

Biological control is a complex process which requires the host to be recognised by the biocontrol agent, followed by hydrolytic enzyme and antibiotic production which is triggered by the direct attachment of the mycoparasite to the host fungus. This contact is mediated by lectins and proteins harboring cellulose binding modules from hyphae of the host and mycoparasitic fungus, respectively, thereby eliciting a signaling cascade comprising G-proteins and MAPKs that can modulate *Trichoderma*’s protein expression pattern [7,8]. However, the detailed molecular mechanisms involved in this process remain unknown. This complex mechanism is influenced by the pathogen and *Trichoderma* isolates evaluated [9]. In this context, studies conducted on different strains are necessary for increased understanding of the biocontrol mechanism.

The sequencing of expressed-sequence-tag (EST) libraries for different *Trichoderma* strains cultivated in the presence of host fungi has contributed significantly to the large-scale identification ofmycoparasitism-related genes [10-12]. Our research group has described gene mapping using EST and suppression subtractive hybridization (SSH) approaches [12,13] during the interaction of *T. harzianum* with *Fusarium solani* and proteomic approaches for *T. harzianum* grown in liquid containing *Rhizoctonia solani*, *Macrophomina phaseolina* and *Fusarium sp* cell walls [14]. Whilst DNA microarrays have been used to study the interaction among *Trichoderma* and host plants [15], only two studies have employed high-throughput transcriptomic approaches to investigate mycoparasitism mechanisms of *Trichoderma*[16,17]. RNA sequencing (RNA-Seq), a high-throughput technology used to sequence complementary DNA, has been widely thought of as a revolutionary tool for transcriptomics [18-21]. The publically available whole genome sequence for *T. harzianum* CBS 226.95 [22], which was recently released by the Joint Genome Institute (JGI) (http://genome.jgi.doe.gov/Triha1/Triha1.home.html), now allows for use of RNA-Seq approaches and mapping of data to the reference sequence, which will likely contribute to identification of mycoparasite-related genes, as well as the molecular mechanisms by which this fungus is able to inhibit phytopathogen fungal growth.

The present study is the first initiative to use RNA-seq for identification of differentially expressed genes in *T. harzianum* strain TR274, in response to the phytopathogenic fungus *S. sclerotiorum. T. harzianum* TR274 was cultivated on liquid medium containing *S. sclerotiorum* cell wall (SSCW) to mimick fungal host presence. Quantitative real-time PCR (RT-qPCR) supported *in silico*-based evidence for differential gene expression of candidate genes involved in mycoparasitism.

## Results and discussion

### Illumina sequencing and mapping onto the *T. harzianum* reference genome

In this present study, an RNA-seq approach was used to map genes differentially expressed during *T. harzianum* growth on SSCW. The experimental design enabled comparison of gene expression in the presence of host cell wall or glucose as sole carbon source. Samples of mRNA from *T. harzianum* following three growth periods in the presence of SSCW (12, 24 and 36 h) were used to construct six Illumina libraries. A total of 171,442,148 sequence reads were obtained after quality trimming, varying from 25 to 100 bp in length. Each library was represented by at least 16,845,349 reads, representing a coverage of 66X when compared with the full transcriptome, a density regarded as adequate to perform gene expression analysis [23]. Complete mapping information can be accessed in Additional file 1: Table S8.

Sequence reads were aligned onto the *T. harzianum* CBS 226.95 reference genome [22]. This strain was isolated from garden soil in the UK, while strain TR274 was isolated from Brazilian cerrado soil. Despite the genetic differences among *T. harzianum* isolates described in the literature [24,4] and the fact that the reference genome published is only a first draft, 82.6% ± 10.08% of the obtained reads were mapped onto the reference genome using the default settings of the Bowtie2 aligner. The high mapping percentage suggests a high similarity between these isolates, at least at the transcriptome level. Only 0.8% ± 0.15% of reads for each library was mapped in more than one region on the reference genome and these reads were not used in the expression analysis.

### Gene expression analysis using “in silico” approach

**Figure 1:**
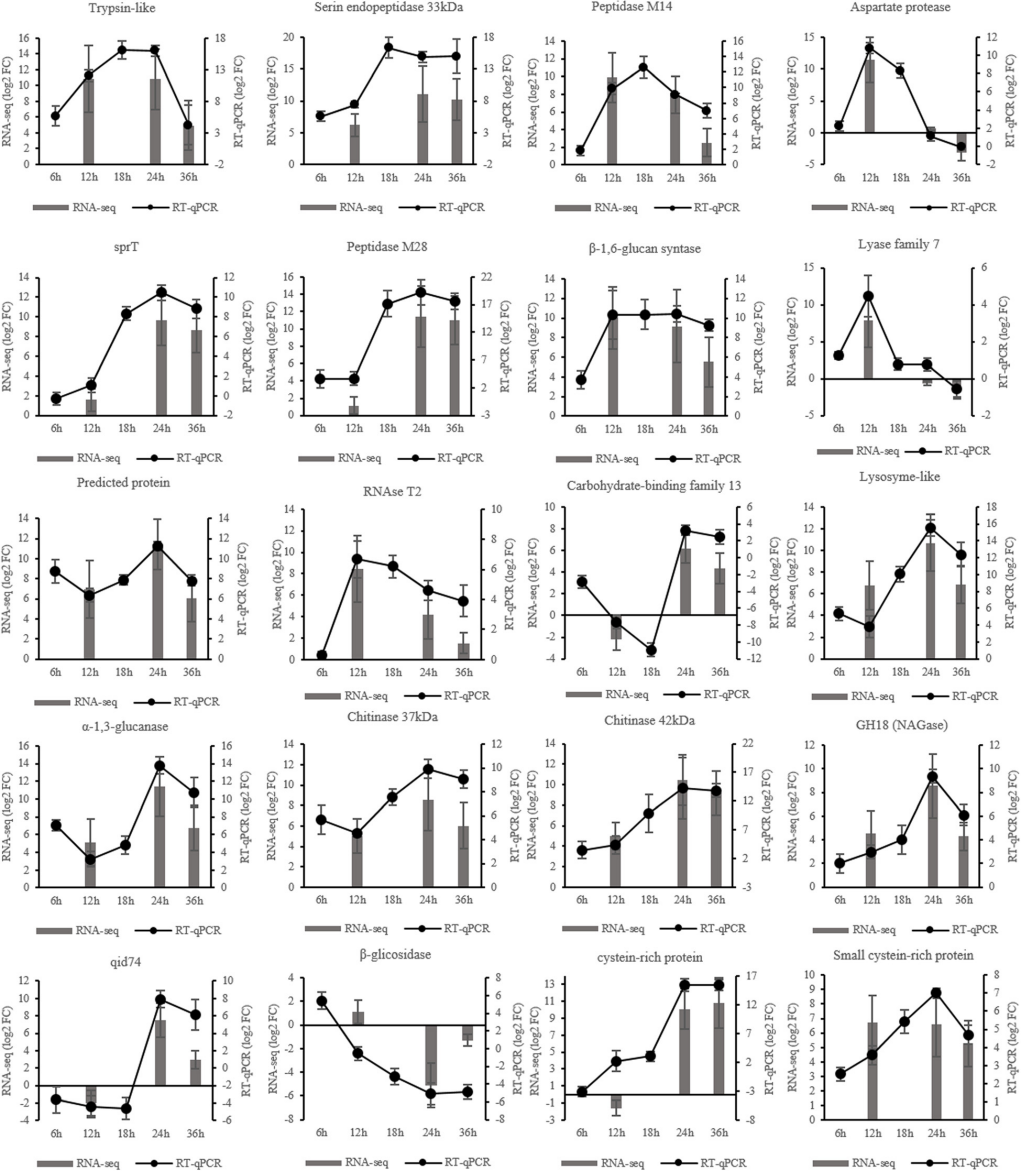
**Comparison of RNA-Seq and real-time RT-PCR analyses.** Histograms represent the relative expression levels (log_2_) (FPKM SSCW/FPKM glucose) as assessed by RNA-seq. Black dotted lines represent expression levels (log_2_) of the relative expression (SSCW compared to glucose for each time) as assessed by RT-qPCR analysis and reported as means and standard errors of three biological replicates for each treatment.

The present work presents a first draft version of *T. harzianum* CBS 226.95 [22] using the RNA-seq approach, and provided a total of 14095 predicted genes. From these, the cuffdiff software analysis detected a total of 297 differentially expressed genes in the presence of SSCW in comparison to glucose as carbon source, as showed by the Venn diagram (Figure 1A). Remarkably, differences in gene distribution pattern were detected when the three induction times were compared. Data suggest a similar expressed gene set distribution between 24 and 36 hours, with most changes detected between 12 hours and 24 hours after *Trichoderma* growth in the presence of SSCW. This same expression pattern was confirmed by gene regulatory network analysis (GNR) (Figure 1B). The main modulation pattern was shared between 12 and 24 hours and a clear inversion between 12 and 36 h. The main genes with different modulation after 12 and 36 h belong to CAZymes and transporters (Additional file 2: Table S1 and Additional file 3: Table S4). This suggests that some proteins required during initial phases of cell wall degradation are not necessary in late times such as 36 hours.

The most notable increase in the gene expression was observed between 12 and 24 hours with a noteworthly change in expression pattern and protein functions detected between 12 and 36 hours. The main genes up or downregulated after 12 and 36 h of growth encoded CAZymes and transporters (Additional file 2: Table S1 and Additional file 3: Table S4). This suggests that some proteins required during early growth phases which are involved in cell wall degradation and sugar transport are no longer necessary after 36 hours of growth.

In order to further evaluate the time course expression profile, the top 10 differentially expressed genes after 12, 24 and 36 hours were identified (Table 1). The top 10 up-regulated genes at 12 h included a chitinase *chi18-17* and a GH25, four proteases, an isotrichodermin c-15 hydroxylase and three proteins of unknown function (Table 1). The top 10 up-regulated genes at 24 h included two peptidases, one Carbohydrate Esterase Family 5 (cutinase), two GH (P1 and α-1,3-glucanase), one transcription factor (srcap-like), two conidiation related and one unknown protein (Table 1). The top 10 up-regulated genes at 36 h included three proteases, a GH76, a MFS transport protein, two proteins involved in cell adhesion (hydrophobin 1 and fasciclin domain protein), an alcohol oxidase, a protease inhibitor kazal and a CBM3 protein. It is interesting to note that there are more proteases/peptidases across the three growth times than GHs in the top 10 up-regulated genes. The combination of proteases and GHs seems to be preferential in mycoparasitism-related conditions [12-14]. Other genes also within the top 10 genes are involved in accessory fuctions like cell adhesion, antibiotic biosynthesis, conidiation and transport, complementing the main degradation activity.

**Table 1:** **log**_**2 **_**Fold Change (FC) of the top 10 differentially expressed genes after 12, 24 and 36 h**

**JGI ID**	**Putative function**	**Top 10**	**12 h**	**24 h**	**36 h**
86893	Aspartic protease	12 h up	11.4208	0.62564	−3.18003
91534	Acid proteinase protein		11.1366	0.04225	−7.00264
526221	Trypsin-like protease		10.8711	10.817	4.95966
154554	Alkaline serine protease		10.7861	0.062284	−8.35379
500888	Chitinase chi18-17		10.7537	−0.26535	−3.95398
521588	Isotrichodermin c-15 hydroxylase		10.6739	6.96978	−1.43531
100207	Hypothetical protein TRIVIDRAFT_62551		10.488	1.96719	1.64245
524327	N,O-diacetyl muramidase(GH25)		10.0928	6.71248	3.93055
43497	Hypothetical protein TRIVIDRAFT_130513		10.0535	6.23974	1.18343
476485	Uncharacterized serine-rich protein		10.0402	2.01486	−0.79684
518894	Peptidase tripeptidyl-peptidase	24 h up	−0.221944	16.3412	21.0713
485240	P1 β-1,6-glucanase		5.2448	11.7243	7.33577
11443	Transcriptional activator srcap-like protein		2.8351	11.5825	8.72928
502174	Conidiation-specific protein (con-13) protein		6.34144	11.5327	4.66579
11439	Hypothetical protein FOC4_g10000877		2.74235	11.4602	8.54921
96797	wsc domain-containing protein		6.99977	11.4475	6.05176
525334	α-1,3-glucanase		5.13055	11.4057	6.72423
501003	Peptidase family m28		1.1649	11.4017	10.9878
128023	Cutinase		0.32008	11.3122	7.58831
503197	Related to spore coat protein sp96 precursor		3.26222	11.1371	9.11624
518894	Peptidase tripeptidyl-peptidase	36 h up	−0.221944	16.3412	21.0713
491972	Fasciclin domain containing protein		0.17976	2.80756	11.4693
501003	Peptidase family m28		1.1649	11.4017	10.9878
96734	Glycoside hydrolase family 76		0.570337	9.93525	10.8109
511478	Proteinase inhibitor kazal		−1.56863	10.0734	10.7725
533861	Hydrophobin 1		−0.624081	9.8541	10.5943
482878	Endonuclease/exonuclease/phosphatase family protein		0.314306	11.0782	10.372
78602	MFS carboxylic acid transport protein		−0.582243	10.0355	10.2595
110777	Alkaline proteinase/serin endopeptidase		6.23477	11.0992	10.226
10644	Alcohol oxidase		6.7904	8.15543	10.0216

**Figure 2:**
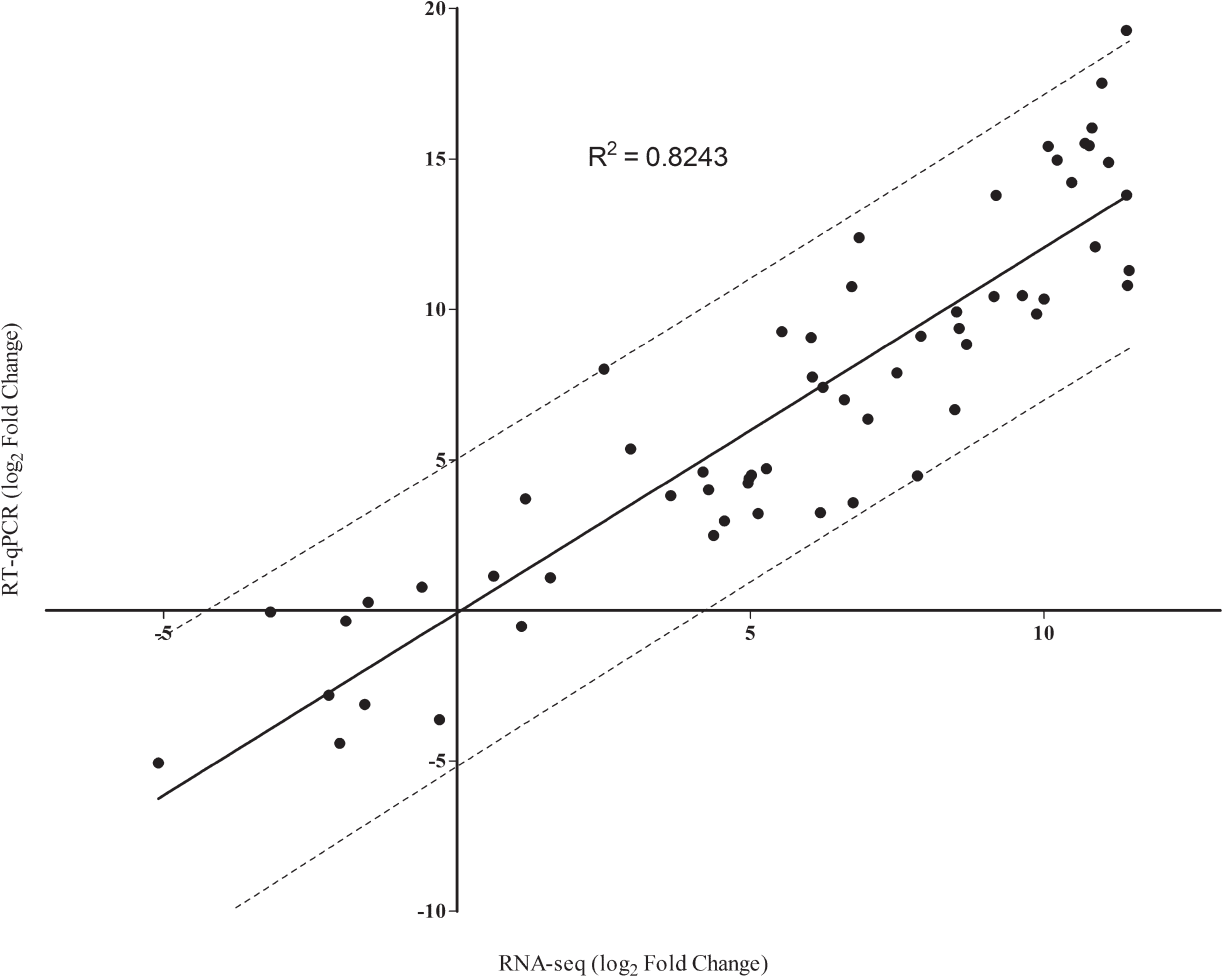
**Pearson correlation between the RNA-seq and RT-qPCR data.** All expression data were normalized in log_2._--- Represents the 95% confidence interval.

To broadly compare gene expression patterns between growth periods, functional categories were assigned to the differentially expressed genes according to Gene Ontology (GO) guidelines [25] using Blast2GO [26]. Interproscan tool was used to improve the Gene Ontology annotations (Additional file 4: Table S2). To enrich the category analysis for up and down regulated genes at each growth time point, an exact Fisher test (p < 0.05) was performed (Figure 2). Data showed a clear up-regulation of transcripts categorized as involved in primary metabolic processes and presenting hydrolase activity, such as chitinases, β-1,3-glucanases and proteases [10,12,13]. This sort of expression pattern is expected, since the host fungus *S. sclerotiorum* cell wall is composed basically of proteins, chitin and β-1,3/1,6/α-1,3glucans [27] and presents the first barrier to be overcome by the mycoparasite to achieve host cell invasion. On the other hand, *Trichoderma* can also use the fungal cell wall as carbon and nitrogen sources, and therefore has to increase expression level of hydrolytic enzymes (chitinases, mutanases, β-1,3-glucanases and proteases) and primary metabolism encoding genes to degrade and metabolize the host cell wall.

The down-regulated transcripts for all the stages of growth were categorized into oxidoreductase activity, oxidation-reduction process and some “binding” child categories, such as small molecule binding and nucleotide binding proteins. A hypothesis for repression could be the nature of basal metabolism of the genes belonging to these categories. This fact is consistent with the extensive metabolic activity expected for a filamentous fungus growing on a rich medium (glucose 2% medium) with an easily assimilable substrate [17]. Under this culture condition up-regulation of a specific subset of oxidoreductases and nucleotide binding proteins endoding genes related to primary metabolism is commonly observed for *Trichoderma* species, which are down-regulated in the presence of complex substrates [13]. Vieira *et al.* in 2013 showed the same pattern of repressed categories when *T. harzianum* was grown on *Fusarium solani* cell wall, suggesting that this result is not pathogen-dependent.

**Figure 3:**
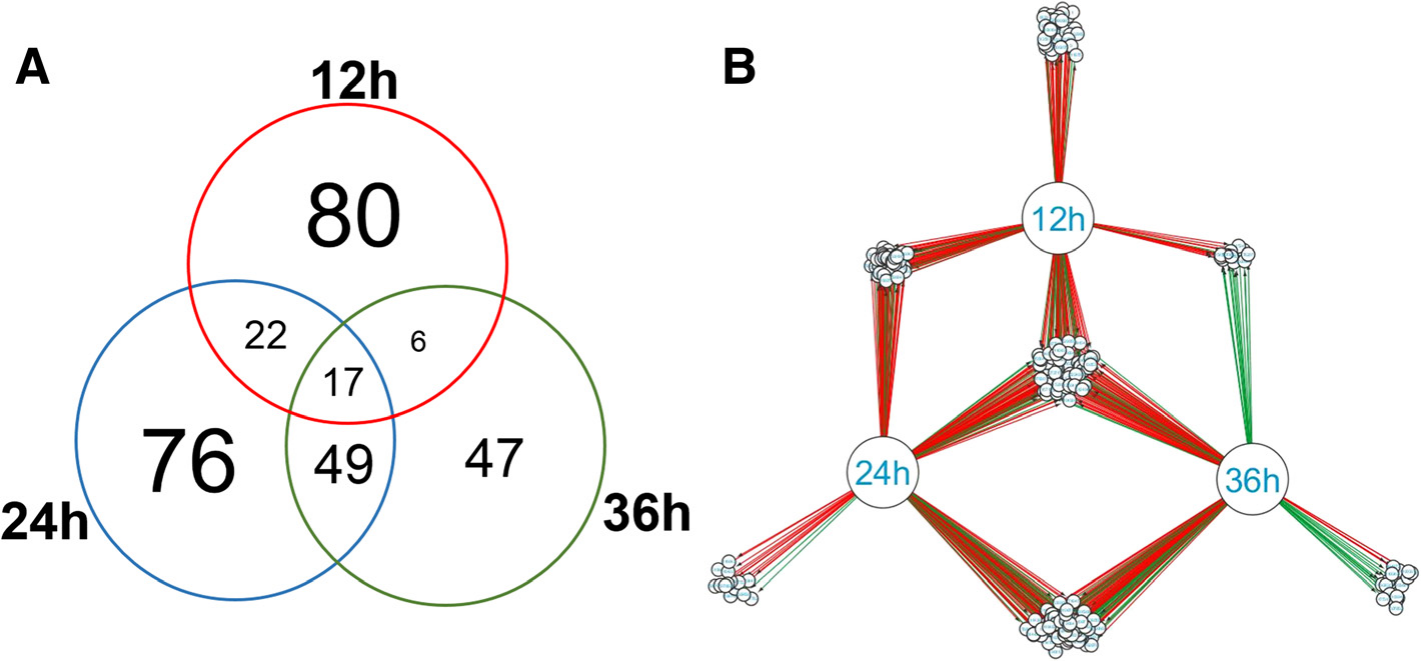
**Venn diagram and network gene interaction of the 297 differentially expressed genes. (A)** Venn diagram showing the distribution of differentially expressed genes detected by cuffdiff (p < 0.05) for *T. harzianum* grown on SSCW for 12, 24 and 36 hours when compared with glucose growth. **(B)** Network gene interaction showing differentially expressed genes up- and down-regulated (Fold Change > 2), for different times (12, 24 and 36 hours) and growth conditions, totalizing 297 genes. Genes are represented as nodes (shown as small circles), and interactions are represented as edges (shown as lines, i.e, red interactions up-regulated and down-regulated interactions green), that connect the nodes: 592 interactions.

Finally, the expression profiles of the differentially expressed genes were determined by cluster analysis based on the SOTA method using Pearson’s correlation distance. These genes were divided into five groups based on their expression modulation over time (Figure 3). Cluster 1 contains genes up-regulated after 12 h growth and down-regulated after 24 and 36 h, cluster 2 contains genes up-regulated after 12 and 24 h and down-regulated after 36 h, cluster 3 contains genes up-regulated during the whole time course, cluster 4 contains genes down-regulated after 12 and up-regulated after 24 and 36 h, and cluster 5 contains genes down-regulated after 12 h and 24 h up-regulated after 36 h. Figure 3A summarizes the clustering analysis as a matrix in which clusters 1 and 2 are presented as mirror images of clusters 4 and 5.

The functional category distribution frequency for each cluster was then calculated to identify differences in the distribution of genes among the three *Trichoderma* growth periods (Figure 3D). High percentages of hydrolases, peptidases and transporter activities were observed in clusters 1, 2 and 3, which included most of the genes up-regulated for all growth times, mainly after 12 h. Clusters 4 and 5 are represented by the lowest number of genes; however they presented a high diversity of functional categories and the smallest percentage of the hydrolase activity category. Cluster 4 is mainly represented by specific transporters, oxidoreductase and peroxidase activities, which are absent in the other clusters. Cluster 5 was less diverse, but contains a high percentage of peptidases and the appearance of the lyase activity category. Clusters 4 and 5 contain genes induced after 24 and 36 hours. This pattern of categorization suggests a late increasing in the expression levels of genes encoding substrate transporters and other CAZYmes such as lyases, instead of the classical cell wall hydrolases (chitinases, β-1,3-glucanases and proteases). Recently, we have described the production of polysaccharide lyases by *T. harzianum* during growth in the presence of different phytopathogen cell walls using a proteomic approach, which is in agreement with the presented data in this work (unpublished data). These results suggest a potential role of these enzymes, especially the lyase families 7 and 8, in mycoparasitism by *Trichoderma* independent of the host pathogen.

### Validation of RNA-seq gene expression

**Figure 4:**
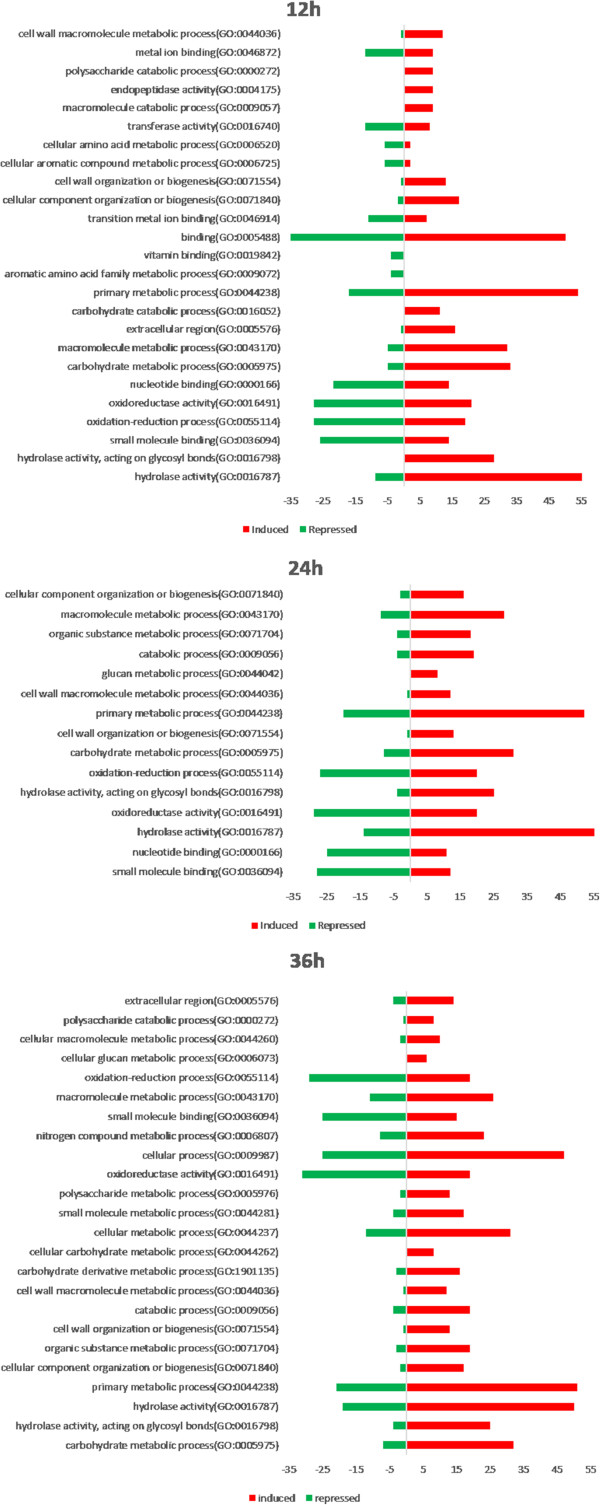
**Annotation of differentially expressed genes.** Distribution of differentially expressed genes using the Blast2GO function prediction tool. All GO functional categories in the differentially expressed genes were compared in the induced (log_2_FPKM > 0) (red) and repressed (log_2_FPKM < 0) (green) genes according to Ficher exact test (p < 0.05).

To validate the expression profile obtained by “*in silico*” analysis of RNA-Seq data, RT-qPCR was performed using 20 genes randomly chosen among up or down-regulated differentially expressed genes. For a better understanding of expression kinetics, two additional growth times were added to the analysis (6 and 18 hours). RT-qPCR expression profiles were in complete agreement with the RNA-Seq data for 17 of the genes evaluated. The other three (Lyase family 7, β-glicosidase, cysteine-rich protein) showed differences at one or two growth times, but the modulation pattern of expression was maintained as observed through the “*in silico*” analysis (Figure 4).

**Figure 5:**
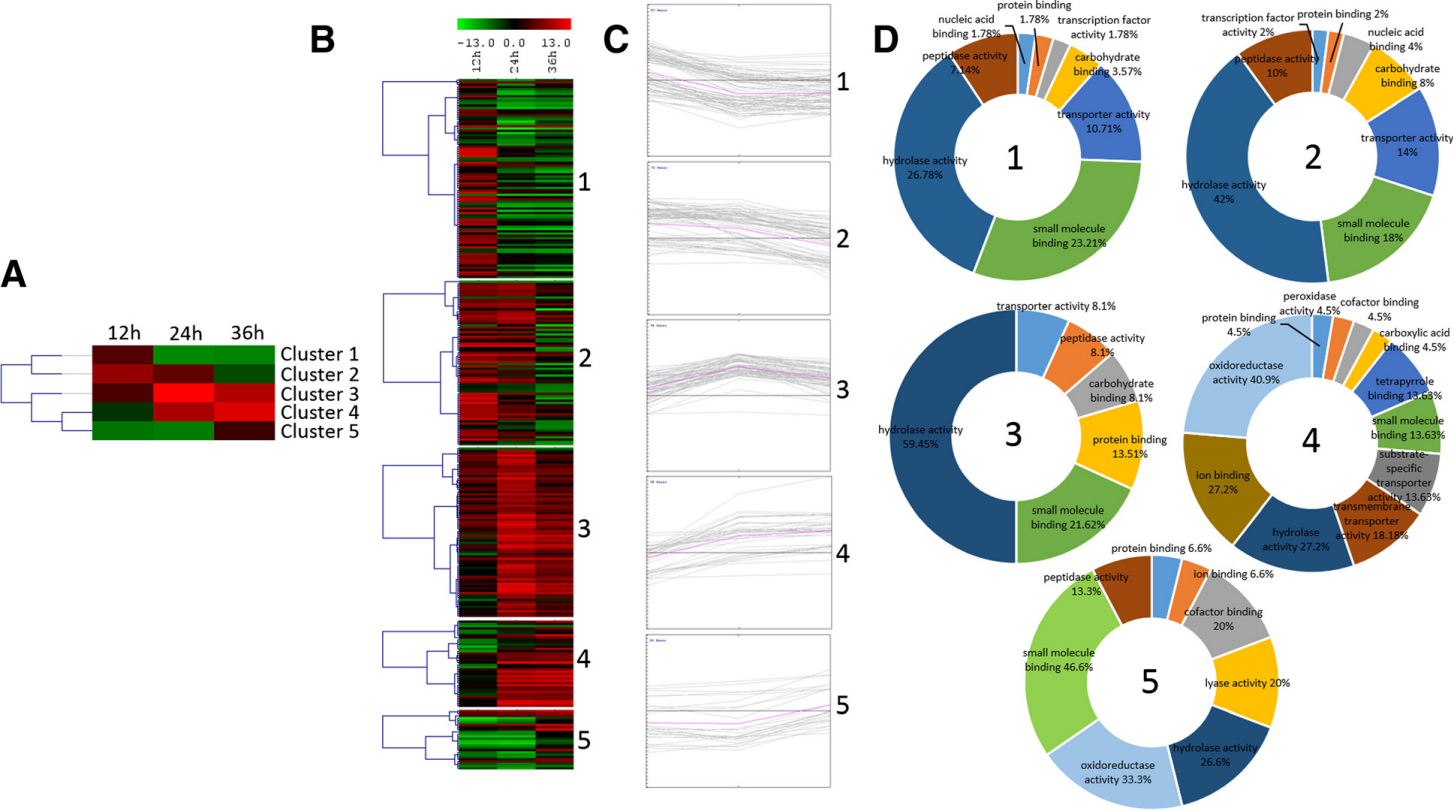
**Heatmap and cluster categorization of the 297 differentially expressed genes showed as log**_**2 **_**FPKM. (A)** Gene expression cluster centroid using SOTA analysis. **(B)** Heatmap of gene clusters **(C)** Clusters expression graphs of differentially expressed genes **(D)** GO functional categories of each cluster. For this categorization only the Molecular Function parent category is presented.

One of the primary goals of transcriptome sequencing is to compare gene expression levels in different samples. In the present work, RNA-seq analysis was carried out for a biological sample of pooled mycelia from three different growth cultures (biological replicates). Validation experiments using qPCR were subsequently carried out using the three biological replicates, and revealed a high Pearson’s correlation coefficient between RNA-seq and qPCR expression data (R^2^ = 0.8243) (Figure 5), this correlation enables us to use this data in differential expression analysis.

As a set of peptidases were up-regulated in the presence of SSCW, two serine peptidases genes (tripsin-like peptidase and serine endopeptidase 33 kDa), one aspartyl protease gene, one aminopeptidase gene (Peptidase M28) and one carboxypeptidase gene (Peptidase M14) were chosen to perform qPCR experiments. The expression data over the five growth times revealed an upward trend along the time course with the highest expression values at 18 hours followed by a decrease after this time or 24 hours. Their diversity and uniform time course production indicated that these enzymes may form a synergistic proteolytic system related to mycoparasistim in *T. harzianum*. However, their exact role in mycoparasitism has not been clearly elucidated yet. The main accepted hypothesis presents these enzymes as contributing to the breakdown of the fungal host cell wall, constituted by chitin and glucan polymers embedded in, and covalently linked to, a protein matrix [28], and also as acting as proteolytic inactivators of pathogen enzymes which are typically involved in the plant infection process [29].

The *Trichoderma* species, *T. reesei, T. atroviride,* and *T. virens*, may have one of the largest sets of proteases among fungi, of which the total number of predicted proteases are 383 (4.2% of all predicted protein coding genes), 445 (3.75%), and 479 (3.85%), respectively [30]. These authors described that the dominant groups are classified as aspartyl proteases, serine proteases, subtilisin-like proteases, and dipeptidyl and tripeptidyl peptidases. These enzymes have been described as performing a central role in the mycoparasitic activity of *Trichoderma* species, as they have been consistently identified during interaction against different phytopathogenic fungi using transcriptomic and proteomic approaches [12-14]. Their diversity and uniform time course production provide evidence that these enzymes may form a synergistic proteolytic system related to mycoparasitism in this *Trichoderma* species. Indeed, in our study, a number of genes encoding a serine peptidase, anaspartyl protease, a subtilisin-like, a trypsin-like beyond metallopeptidases (M28, M14) were also differentially expressed in the presence of SSCW. These results strongly suggest a role of these enzymes in *T. harzianum* mycoparasitism against *S. sclerotiorum*, and support a putative common action mechanism of mycoparasite fungi within the *Trichoderma* genus.

About 29% of the differentially expressed genes encode CAZy enzymes (Additional file 2: Table S1), suggesting a central role for them in Trichoderma mycoparasitism, probably during host fungal cell wall degradation. The kinetics expression for CAZy category enzymes presented as expression average showed a decrease trend along the growth timecourse, with a maximal expression at 24 h. An exception were the enzymes categorized as auxiliary activities and polysaccharide lyases (Additional file 5: Table S3). Glycosyl hydrolase family 18 and other enzymes which act mainly as fungal cell wall degrading enzymes (CWDE) have also been described as presenting a central role in mycoparasitism in *Trichoderma atroviride*[31]. Genes encoding this family were also up-regulated in the present work based on “*in silico*” RNA-seq data analysis. As a consequence, eight genes encoding three GH18 (Chitinase 37 kDa, Chitinase 42 kDa and a not well characterized Chitinase), a α-1,3-glucanase, a β-glicosidase, a lyzosyme-like, a polyssacharide lyase Family 7 and a carbohydrate binding Family 13 protein were selected for expression validation by RT-qPCR. The expression levels of all CWDE genes followed the same trend, except for the β-glucosidase gene that was repressed from 12 hours onwards. The common expression profile was an increase in transcripts after 12 hours until 24 hours and a similar level or decrease of transcripts at 36 hours. This kinetic suggests use of small sugars during the first growth time and an expression of CWDE after 12 hours, indicating a role in degradation of the cell wall as carbon source to allow continued growth. KEGG pathway analysis (Additional file 6: Table S7) shows the mapped genes in starch and sucrose metabolism. All genes mapped are in the final stages of the pathway, mainly in the formation of small sugars such as D-glucose and D-xylose.

Alginate lyases are enzymes classified as belonging to the polyssacharide lyase Family 7 and are usually involved in the deconstruction of complex polyssacharides, such as polyguluronate and polymannuronate [32]. Their expression observed in our study suggests a possible role in mycoparasitism, complementing the classical GH activity which is known to be involved. The carbohydrate binding module Family 13 gene encodes a protein with a domain related to lectins, which, in *Rhizoctonia solani*, is implicated in fungal insecticidal activity [33]. Our expression data suggest that this gene is related to the lectins and may play a role mediating the physical contact with the host and elicitation of the signaling cascade comprising G-proteins and MAPKs.

Small secreted cysteine-rich proteins (SSCPs) have been described as up-regulated in *Trichoderma* species during mycoparasitism against phytopathogenic fungi [31]. The present work is the first to report their probable role in mycoparasitism of *T. harzianum* against *S. sclerotiorum*.

In this work we also indentified genes enconding two predicted cistein-rich proteins and *qid74*, all up-regulated in the presence of SSCW. These genes were highly expressed in the later induction time periods (24 and 36 h) as showed by “*in silico*” analysis of RNA-seq and are in agreement with the results of validation by RT-qPCR. Hydrophobins I and II were also identified by the “*in silico*” analysis of RNA-seq data, as shown in Additional file 4: Table S2 with a high expression at 24 and 36 hours.

Small secreted cysteine-rich proteins (SSCPs) are one of the largest groups of proteins secreted by *Trichoderma*. Hydrophobins, probably the most widely known SSCPs, are characterized by the presence of eight positionally conserved cysteine residues of which four occur in doubles. They are found on the outer surfaces of cell walls of hyphae and conidia, where they mediate interactions between the fungus and the environment and also between the fungus and host plant roots [34]. Class II hydrophobins represent the predominant class described for *Trichoderma* species [35]. *T. atroviride* and *T. virens* have also class I-like hydrophobins, however they present differences in several aspects when compared to other fungi and form a separate clade in phylogenetic analysis within the Ascomycetes [36]. As well as other cysteine-rich proteins, *T. harzianum* the *qid74* gene encodes a cell wall protein which has an important role in adherence to hydrophobic surfaces and cellular protection [34]. The gene RNaseT2, which has been described as a stress related protein and involved in permeability and stability of the plasmatic membrane in *Saccharomyces cerevisiae*[37]*,* was also up-regulated after 12 hours growth of *T.harzianum* on SSCW, decreasing over time.

Among the 297 differentially expressed genes, 30 encode transporter proteins (Additional file 3: Table S4). MFS (Major Facilitator Superfamily) permeases are the most abundant proteins among transporter proteins over the three growth times. These proteins enable the transport of essential nutrients and ions, in addition to the excretion of end products of metabolism and cell-environment communication [38]. Their expression levels vary according to the time of growth and culture growth condition (presence or absence of SSCW and glucose).

In summary, our results demonstrated a time course dependent expression of *T. harzianum* genes during growth on media with *S. sclerotiorum* cell wall as sole carbon and nitrogen source. The majority of the genes described in this work have also been reported in the literature during growth of *Trichoderma* spp. on cell-wall of phytopathogenic fungi [10,12,13], as well as during confrontation against *R. solani*[31].

### RT-qPCR for dual cultures

**Figure 6:**
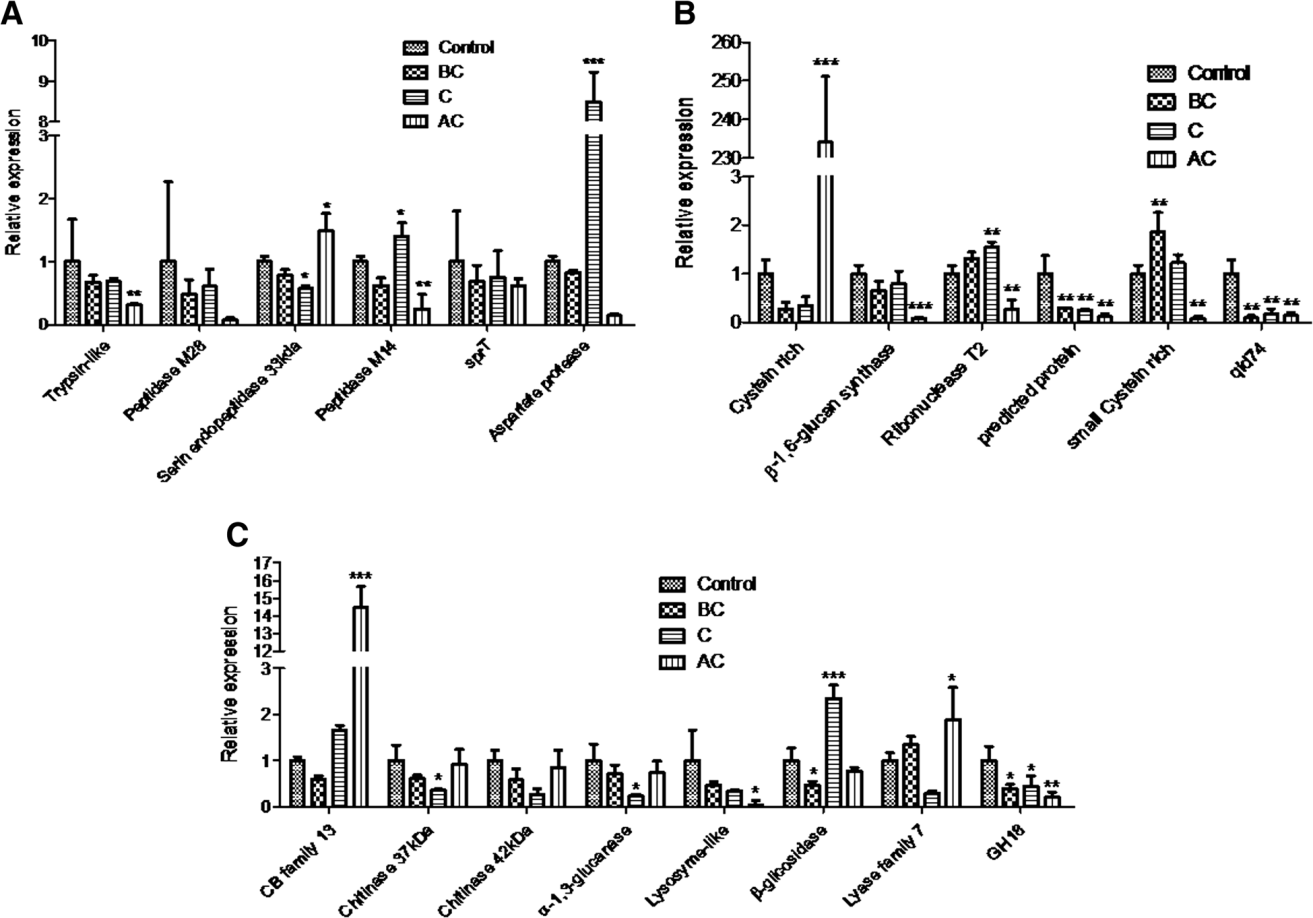
**Differential expression analysis and quantification of transcript levels of biocontrol-related genes expressed by *T. harzianum *under mycoparasitic conditions asgainst *S. sclerotiorum*.** Control, BC – Before contact, C – Contact, AC – After Contact. **(A)** Expression analysis of Trypsin-like protein, Peptidase M28, serin endopeptidase 33 kDa, Peptidase M14, sprT, Aspartate protease. **(B)** Expression analysis of cystein rich protein, β-1,6-glucan synthase, ribonuclease T2, predicted protein, small cysteine rich protein, qid74. **(C)** Expression analysis of carbohydrate binding module family 13 protein, chitinase 37 kDa, Chitinase 42 kDa, α-1,3-glucanase, lysozyme-like protein, β-glicosidase, lyase family 7 protein, Glycoside Hydrolase family 18 protein. The data were presented as fold change using the 2^-ΔΔCt^ method.* p<0.05, ** p<0.01, ***p<0.001.

The direct confrontation assay is a powerful tool for studyng mycoparasitism by *Trichoderma*[12,13,30] under laboratory conditions. To validate RNA-seq data using a growth condition which closely mimics the interaction *Trichoderma*/host fungus, RT-qPCR was also performed using total RNA from dual cultures of *T. harzianum* and *S. sclerotiorum* over three different interaction stages: before physical contact (BC) between the mycoparasite and the host, during the contact (C) and after the contact (AC). As a control, a confrontation assay was conducted in which *T. harzianum* was challenged against itself. The same genes chosen for the RNA-seq “*in silico*” analysis validation were used in this analysis, with three genes presenting expression patterns in agreement with RNA-seq data (Figure 6). The peptidases serine endopeptidase, M14 peptidase (carboxypeptidase) and aspartate protease were up-regulated during and after contact between *T. harzianum* and *S. sclerotiorum,* when compared with the control bioassay, providing further evidence for these enzymes as important factors in the mycoparasitism (Figure 6A).

The two small cystein-rich proteins were up-regulated during the interaction between *T. harzianum* and *S. sclerotiorum* although they showed differing expression patterns over the time period (Figure 6B). The gene cystein rich (511478) was highly expressed after contact, whilst the gene small cystein rich (518220) before contact. These data suggests that this group of proteins (SSCP) may present a synergistic time course dependent activity during the interaction. The RNAse T2 gene was also up-regulated during the contact stage, confirming its role in this interaction, possibly through conferring membrane stability during contact with the phytopathogen hyphae (Figure 6B).

Among the eight genes encoding putative glycoside hydrolases, three were up-regulated in the interaction: polyssacharidelyase Family 7, β-glicosidase and the CB module Family 13. This data suggests a role of these proteins in the mycoparasitism, complementing the classical GH activity in the interaction. The carbohydrate binding module Family 13 gene was induced during and after contact with *S. sclerotiorum*. The other genes identified as up-regulated by RNA-seq “*in silico*” analysis and validated by qRT-PCR using SCCW were not up-regulated in this condition. This “not-complete” agreement between cell wall induction with SSCW and direct confrontation with *S. sclerotiorum* is expected due to differences in growth conditions for *T. harzianum*. Investigation of mycoparasitisn using inactivated cell walls is a useful approach to evaluate the broad arsenal of induced genes in *Trichoderma spp.* and to identify candidate mycoparasitism related genes that can be further evaluated for expression during mycoparasitism through dual culture assays. The genes which were observed to be up-regulated in both interaction bioassays are certainly promising candidates for future biotechnological applications as well as further detailed investigations to unveil their precise function in *T. harzianum* mycoparasistism.

## Conclusions

The RNA-seq data presented will not only facilitate improvement of the annotation of gene models in the draft *T. harzianum* genome, but also provide important information regarding the transcriptome during growth on SSCW and during “*in vivo*” interactions with *S. sclerotiorum*. Our data represent an important step towards understanding the mycoparasitic process of *T. harzianum* during its interaction with *S. sclerotiorum.* Further studies for functional characterization of candidate genes reported here are necessary in order to better define the exact pathways involved in mycoparasitism in *T. harzianum.*

## Methods

### Organism culture conditions

*T. harzianum* TR274 was isolated from soil samples from the Brazilian Cerrado region and identified to species level based upon ribosomal RNA ITS 1 and 2 sequence identities (Genbank number KC993076). This strain is deposited in the ICB Enzymology Group culture collection at the Universidade Federal de Goias. A strain of the phytopathogenic fungus *S. sclerotiorum* belonging to the EMBRAPA-CNPAF culture collection was originally isolated from *Phaseolus vulgaris*. These fungi were grown on MYG medium (w/v: 0.5% malt extract, 0.25% yeast extract, 1% glucose and 2% agar) for 2 days at 28°C. *T. harzianum* spores were collected from cultures by washing with sterile water and centrifugation at 2000 rpm. Following two rounds of washes, spore suspensions at a concentration of 10^7^ spores mL^−1^ were used to inoculate flasks containing 50 mL of TLE medium [14]. Cultures were incubated at 28°C with constant shaking at 120 rpm. After 24 hours growth, mycelia were collected and transferred to flasks containing 50 mL of minimal medium (TM) (w/v: 0.2% KH_2_PO_4_, 1.4% (NH_4_)_2_SO_4_, 0.03% MgSO_4_.7H_2_O) supplemented with 2% (w/v) of glucose or 0.5% (w/v) of inactivated SSCW (previously autoclaved at 120°C for 20 min). Cultures were incubated with constant shaking at 120 rpm at 28°C. After 6, 12, 18, 24 and 36 hours of growth, mycelium was harvested and immediately flash frozen in liquid nitrogen and stored at -80°C until RNA isolation. This experiment was carried out in triplicate for each growth period, with mycelia subsequently pooled to constitute composed samples.

### RNA isolation, cDNA library preparation and sequencing

RNA was extracted from macerated frozen mycelia using TRI-Reagent (Sigma-Aldrich), according to manufacturer’s instructions. Integrity and quantity of isolated RNA were assessed using a RNA Pico chip on an Agilent Bioanalyzer 2100 (Agilent Technologies) (Additional file 7: Table S5). The RNA samples were stored at -80°C until library construction for sequencing and RT-qPCR. Normalized starting quantities of total RNA extracted from mycelia grown for 12, 24 and 36 h under the two different conditions (supplemented with glucose or SSCW), were then used to prepare six separate barcoded RNA-seq libraries using the TruSeq™ RNA sample preparation kit (Illumina, CA, USA). Three biological replicates were pooled in preparation of each of the six final samples. All library preparation and sequencing was carried out by Eurofins MWG Operon (Al, USA). Barcoded libraries were prepared according to the manufacturer’s instructions, then sequenced on a single lane on an Illumina HiSeq2000 sequencer, to generate 100 bp paired-end reads.

### Mapping of sequenced reads and assessment of gene expression

FastQCfiles were used to visualize the libraries quality before and after trimming. For quality trimming and sequence filtering, the software Trimmomatic (version 0.30) [39] was employed to remove sequencing adapters, k-mers, and bases with a Phred quality score lower than 20 from the read ends. All reads shorter than 25 nucleotides were then discarded. Filtered reads were mapped onto the *Trichoderma harzianum* v1.0 genome sequence (http://genome.jgi.doe.gov/Triha1/Triha1.home.html) using TopHat 2.0.8 release with default settings [40]. Gene expression values were determined using Cufflinks 2.1.1 release [41], and the FPKM (Fragments per kilobase mapped) values were calculated for each transcript. The gene expression levels in the *T. harzianum* genome were obtained using the Cuffdiff software within Cufflinks. Fungal transcript levels were calculated using uniquely mapped reads onto the genome. The expression profiles of differentially expressed genes were determined by SOTA (Self Organizing Tree Algorithm), cluster analysis was carried out using the MeV 4.9 software, and gene ontology assignment conducted according to Gene Ontology (GO) guidelines [25] using the Blast2GO platform [26].

Each *T. harzianum* sample grown in SSCW had a corresponding control sample of culture growth on glucose, enabling normalization using the relation FPKM SSCW/FPKM glucose. Positive values were considered as up-regulated transcripts in the presence of cell wall and negative values were considered as down-regulated transcripts. Expression level differences were judged to be significant and the expression level estimate status acceptable when a gene was identified as differentially expressed with an FDR of the Benjamini-Hochberg multiple tests of 5% (P < 0.05).

### Gene regulatory network of *T. harzianum*

A regulatory network was generated using Cytoscape 3.0.2 software and a table of data containing the differentially expressed genes detected using the cuffdiff program, presenting Fold Change > 2, the interaction type (up- or down-regulated) and the target gene (i.e., the Protein ID of each gene affected). This analysis was carried out in order to reconstruct a *T. harzianum* time course network representation for all 297 identified genes (Figure 1B) [42].

### RT-qPCR

Twenty genes were randomly selected between the differentially expressed genes *in silico* for the expression analysis by RT-qPCR. Real-time qPCR (Additional file 8: Table S6) primers were designed using the PerlPrimer v1.1.20 software. Total RNA from the above described preparations was digested with DNase I (Invitrogen) and a total of 5 μg from each pooled sample was reverse transcribed into cDNA using the Maxima™ First Strand cDNA synthesis kit for RT-qPCR in a final volume of 20 μL (Fermentas). The synthesized cDNA was diluted with 80 μL of water and used as a template for real-time PCR reactions using the instrument iQ5 real-time PCR system (Bio-Rad). Each reaction (20 μL) contained 10 μL of MAXIMA® SYBR-green PCR Master Mix (Fermentas), forward and reverse primers (500 nM each), cDNA template, and nuclease free water. PCR cycling conditions were: 10 min at 95°C (1 cycle), 15 s at 95°C followed by 1 min at 60°C (40 cycles), and a melting curve ramping from 60°C to 95°C with an increasing temperature of 0.2°C for 10 s (1 cycle) and continuous data collection to test for primer dimers and nonspecific amplification. Determination of the PCR efficiency was performed using triplicate reactions from a dilution series of cDNA (1, 0.1, 10^−2^, and 10^−3^). Amplification efficiency was then calculated from the given slopes in the IQ5 Optical System Software v2.0 (Additional file 8: Table S6). The α-tubulin and β-actin transcript were used as internal references to normalize the amount of total RNA present in each reaction [12]. Gene expression levelswere calculated from the threshold cycle according to the 2^-ΔΔCT^ method [43]. All samples were analyzed in at least two independent experiments with three technical replicates in each run.

### Analysis of expression of biocontrol-related genes

RT-qPCR was used to evaluate *T. harzianum* gene expression during confrontation against the fungal pathogen *S. sclerotiorum*. Confrontation bioassays were conducted as described in Steindorff *et al*. [12]. *T. harzianum* TR274 and *S. sclerotiorum* circular plaques of 5 mm diameter were cut from 7-day-old cultures on MYG Plates. *T. harzianum* TR274 plaques were inoculated onto plates containing solid minimal medium supplemented with 0.2% of glucose and overlaid with cellophane at a distance of 7 cm from *S. sclerotiorum* plaque mycelia. A control confrontation assay was conducted following the same setup described above, except that *T. harzianum* was challenged against itself. Confrontation plates were incubated in the dark at 28°C and the mycelia were harvested at different growth stages: prior to fungal contact, at contact, and after contact (overlapping mycelia). The confrontation assays were conducted in triplicate with RNA extracted for all treatments and replicates. The RNA samples were used for RT-qPCR reactions as described above, with results compared by ANOVA coupled with the Dunnet’s test (α = 5%) using GraphPad Prism 5 software, to allow analysis of differences between confrontation assay gene expression patterns.

### Availability of supporting data

Sequences have been deposited at the Sequence Read Archive (SRA) of the National Center for Biotechnology under BioProject number PRJNA216008. Raw sequence reads can be found in http://www.ncbi.nlm.nih.gov/sra/?term=PRJNA216008.

## Competing interests

The authors declare that they have no competing interests.

## Authors’ contributions

ASS, GJP, ASGC, RNGM, EFN performed the experimental design, RNA isolation, quality control and designed the bioinformatics analysis. ASS and MHSR performed the RT-PCR analyses and evaluation of the data. ASS drafted the manuscript. EFN and RNGM were responsible for revision of the manuscript. All authors approved the final version of the paper.

## Supplementary Material

Additional file 1: Table S8 KEGG pathwayanalysisshownenzymesmappedin StarchandSucroseMetabolism. Arrowsin red, greenandblue (12, 24 and36h respectively) show theup-regulatedconditionofallgenes. Click here for file

Additional file 2: Table S1 Primers used in qPCR experiments. Click here for file

Additional file 3: Table S4 CAZy enzymes differentially expressed after 12, 24 and 36 h. Click here for file

Additional file 4: Table S2 RNA-Seq sequencing and read mapping. Click here for file

Additional file 5: Table S3 Functional annotation of the 297 differentially expressed genes. Click here for file

Additional file 6: Table S7 log2 Fold change expression of CAZy classes. The data shown is the average and standard deviation on each time condition. Click here for file

Additional file 7: Table S5 Transporters differentially expressed in 12, 24 and 36 hours. Click here for file

Additional file 8: Table S6 Bioanalyser profile of the six samples used to construct RNA-seq libraries. Click here for file
