# Supplementary material for: Identification of mycoparasitism-related genes against the phytopathogen Sclerotinia sclerotiorum through transcriptome and expression profile analysis in Trichoderma harzianum
Source: BMC Genomics. 2014 Mar 18;15:204. doi: 10.1186/1471-2164-15-204 (PMC4004048; doi:10.1186/1471-2164-15-204)
Supplement: Additional file 2: Table S1 — Primers used in qPCR experiments. [file 1471-2164-15-204-S2.pdf]

Additional table 1 – Primers used in qPCR experiments

			Primers for qPCR (5' to 3')	
**Putative function**	**JGI id**	**Efficiency (%)**	**Forward**	**Reverse**
cystein rich protein	511478	104.4	ACTGCGGCATGGCATGTGATATTC	AACAAATACCACCGCAGTCTGCAC
Carbohydrate-binding module 13	511345	100.2	TTCACGGGAACAGGTCAACACCTA	TTTGAAGGAACCAGAGCCCAATGC
Trypsin-like protein	526221	109.5	ACCCTGGTTGGTGTTGTTTCTTGG	TGATGAAGCTGACGTAGTTGCCCA
Chitinase 37 kDa	505895	101.2	AGGCACCACAATCAAGGTTCCCTA	TTGGACCCATCCCAGTTGATTGAC
β-1,6-glucan synthase	487382	108.5	GAAGGATTTCGGTGCCAGCATTGT	TGGACAATCAGGCCCATGTTGTTG
Peptidase M28	501003	102	GWCTTCTTGGCTCTCAGGCAATCT	GTCAGTATAGACAGCCAGCTGGTT
Serin endopeptidase 33kDa	110777	100.2	AATGACCTCCACCGTCGTTCTCTT	ATGTAGTCTGGCTCAACGGAAGCA
Chitinase 42 kDa	101028	109.5	TCAACACCAAGGTTGCCTACCTCA	TCAGCAGGTTCTGAGTTGTGTCCA
Peptidase M14	514267	107.3	GATCTGAACCGCAACTTCGACTTC	TGAAGGTCGATGAACCAGGCAATC
α-1,3-glucanase	525334	108.6	GTCTGGATGTAAATGCGTTGCGCT	TCCATCATTATCCCAGGCCATCCA
sprT	511032	109.6	AACTACGGCTCCGTCGTGGATATC	GGAGTTGCCATGGAGGTACCAGA
lysosyme-like	503484	98.3	ATCAACCAATCTGGCCGCAGTGAT	CAATATCACTGGCGTAGCAGTGAG
Aspartate protease	86893	100.4	GACACAGTTAGCTTCGGTGGTTTG	GATCCACCGGACCAAAGCCAATAA
β-glicosidase	127782	100.1	ACTTACGACCATCAACCAGCACGT	ATGACGGCAATGCTCTTGGGTTTC
Lyase family 7	619	99.4	AATTCGGCGAGACTCAATCGTGGA	TGCCGTTGTAGTAAACCTCAGCGA
Ribonuclease T2	153694	99.4	ACCAACTATCAGCCAACCCAGGAA	AGCCTGGATAGCAGCCAAAGTGTA
Glycosyde hydrolase family 18	509593	99.7	ACTTTGGCCCAGACTTCCTCATCA	TACCAGTCAATGTTGGAGGCCTGT
Predicted protein	96797	99.2	TTGCCTTCCCTGCTATTGGTGACT	TGGCGTAGACGAATCGGTTGAAGT
Small cystein-rich protein	518220	96.2	TTGGCAACTTCCGATCCCAGACTA	TGTGGAACCAGTAAGCCGGGAAAT
qid74	509414	92.1	AAGCAGGACATCAAGAAGGGCAAG	AAGGCACCGTAGTTCCAGAGGTA
act1	84596	95.2	GACATTTACGGCGGAGATGAG	AGGACGGCAGGATTGATTTG
α-tubulin	76895	102.5	TATCTGCTACCAGGCTCCCGAGAA	TGGTGTTGGACAGCATGCAGACAG
