# Supplementary material for: Identification of mycoparasitism-related genes against the phytopathogen Sclerotinia sclerotiorum through transcriptome and expression profile analysis in Trichoderma harzianum
Source: BMC Genomics. 2014 Mar 18;15:204. doi: 10.1186/1471-2164-15-204 (PMC4004048; doi:10.1186/1471-2164-15-204)
Supplement: Additional file 3: Table S4 — CAZy enzymes differentially expressed after 12, 24 and 36 h. [file 1471-2164-15-204-S3.docx]

Additional file 3: Table S4 – CAZy enzymes differentially expressed after 12, 24 and 36h

| **JGI ID** | **Putative Function** | **Class** | **CAZy Family** | **12h** | **24h** | **36h** |
| --- | --- | --- | --- | --- | --- | --- |
| 510295 | multicooper oxidase (laccase) | Auxiliary Activities | 1 | 0 | 6.46552 | 6.8291 |
| 523580 | catalase-peroxidase 2 | Auxiliary Activities | 2 | -1.82727 | -4.67979 | -4.0495 |
| 10644 | alcohol oxidase | Auxiliary Activities | 3 | 6.7904 | 8.15543 | 10.0216 |
| 512860 | glucose-methanol-choline oxidoreductase | Auxiliary Activities | 3 | -5.84819 | -1.83222 | 1.83709 |
| 510452 | berberine family protein | Auxiliary Activities | 7 | 3.80511 | 5.16515 | 6.89997 |
| 126387 | fad binding domain-containing protein | Auxiliary Activities | 7 | -0.1673 | 8.32345 | 5.32994 |
| 484109 | Expansin-like protein | Carbohydrate Binding Module | 1 | 7.41261 | 4.14586 | 2.01604 |
| 155649 | catechol dioxygenase | Carbohydrate Binding Module | 12 | 6.733 | 5.84819 | 6.83111 |
| 501003 | peptidase family m28 | Carbohydrate Binding Module | 12 | 1.1649 | 11.4017 | 10.9878 |
| 9084 | Carbohydrate-binding protein 13 family protein | Carbohydrate Binding Module | 13 | 0.95751 | 5.82292 | 4.2840 |
| 511032 | subtilisin-like protease pr1a | Carbohydrate Binding Module | 13 | 1.58997 | 9.6325 | 8.6792 |
| 110777 | alkaline proteinase | Carbohydrate Binding Module | 2 | 6.23477 | 11.0992 | 10.226 |
| 482878 | Endonuclease/exonuclease/phosphatase family protein | Carbohydrate Binding Module | 3 | 0.3143 | 11.0782 | 10.372 |
| 99712 | carbohydrate-binding protein | Carbohydrate Binding Module | 32 | 8.09646 | 4.18577 | 1.53130 |
| 526221 | trypsin-like protease | Carbohydrate Binding Module | 5 | 10.8711 | 10.817 | 4.95966 |
| 124624 | hypothetical protein TRIVIDRAFT_201746 | Carbohydrate Binding Module | 50 | 5.8152 | 5.90926 | 5.84215 |
| 514267 | peptidase m14 | Carbohydrate Binding Module | 50 | 9.87753 | 7.90271 | 2.5041 |
| 118590 | hypothetical protein TRIATDRAFT_46148 | Carbohydrate Binding Module | 50 | 8.40114 | 0.206307 | -0.57087 |
| 69921 | chitinase-like protein | Carbohydrate Binding Module | 50 | 8.63973 | 2.92718 | 1.54577 |
| 513561 | hypothetical protein TRIATDRAFT_58828 | Carbohydrate Binding Module | 32 | 6.60082 | 1.26395 | -0.05037 |
| 102504 | carbohydrate esterase family 1 protein | Carbohydrate Esterases | 1 | 2.17465 | 8.18953 | 4.01678 |
| 103604 | triacylglycerol lipase | Carbohydrate Esterases | 10 | 6.24728 | 3.17359 | 2.74681 |
| 17178 | alpha beta hydrolase fold protein | Carbohydrate Esterases | 10 | -2.24196 | -7.57032 | -7.28131 |
| 525304 | Cyclopentanone 1,2-monooxygenase | Carbohydrate Esterases | 10 | 6.48347 | 7.60762 | 5.17966 |
| 9252 | carboxylesterase | Carbohydrate Esterases | 10 | 11.1366 | 0.04225 | -7.00264 |
| 511858 | aldehyde reductase ii | Carbohydrate Esterases | 12 | -0.899917 | 8.07985 | 8.56271 |
| 13053 | nad dependent epimerase dehydratase | Carbohydrate Esterases | 12 | 5.88954 | 9.57783 | 6.21592 |
| 507869 | nad dependent epimerase protein | Carbohydrate Esterases | 12 | -0.474467 | 5.66127 | 3.05484 |
| 494647 | carbohydrate esterase family 15 | Carbohydrate Esterases | 15 | 0 | 6.89479 | 1.43187 |
| 18043 | polysaccharide deacetylase family protein | Carbohydrate Esterases | 4 | 5.93377 | 6.85415 | 2.49124 |
| 128023 | cutinase | Carbohydrate Esterases | 5 | 0.32008 | 11.3122 | 7.58831 |
| 118643 | acetyl xylan esterase | Carbohydrate Esterases | 5 | 2.90133 | 9.91574 | 0.09433 |
| 525334 | glycoside hydrolase family 71 protein | Glycoside Hydrolases | 71 | 5.13055 | 11.4057 | 6.72423 |
| 122717 | NmrA-like protein | Glycoside Hydrolases | 10 | -5.77568 | -8.21736 | -7.76556 |
| 91773 | glycosyl hydrolase family 10 protein | Glycoside Hydrolases | 10 | 0.10043 | 8.69453 | 0.32073 |
| 112585 | glycoside hydrolase family 12 protein | Glycoside Hydrolases | 12 | 8.51887 | 5.22419 | 0.218219 |
| 476485 | uncharacterized serine-rich protein | Glycoside Hydrolases | 128 | 10.0402 | 2.01486 | -0.796843 |
| 487382 | β-1,6-glucan synthase | Glycoside Hydrolases | 17 | 10.0017 | 9.14618 | 5.53615 |
| 97878 | gpi anchored cell wall protein | Glycoside Hydrolases | 17 | -7.53964 | -0.3380 | 1.00627 |
| 509536 | ww domain-containing oxidoreductase | Glycoside Hydrolases | 18 | -0.90944 | -7.66647 | -2.86409 |
| 101028 | endochitinase 42 | Glycoside Hydrolases | 18 | 4.97365 | 10.4723 | 9.1817 |
| 91651 | hypothetical protein TRIATDRAFT_43673 | Glycoside Hydrolases | 18 | -2.0299 | 7.25928 | 4.07314 |
| 45593 | glycoside hydrolase family 18 protein | Glycoside Hydrolases | 18 | 7.48019 | 0.91216 | 0.34228 |
| 505895 | chitinase 37kDa | Glycoside Hydrolases | 18 | 5.01951 | 8.51308 | 6.03478 |
| 476542 | hypothetical protein | Glycoside Hydrolases | 18 | 2.12725 | -7.23224 | -5.87795 |
| 509593 | glycoside hydrolase family 18 protein | Glycoside Hydrolases | 18 | 4.55401 | 8.5532 | 4.28326 |
| 477022 | ww domain-containing oxidoreductase | Glycoside Hydrolases | 18 | -6.45518 | -7.0922 | -6.10829 |
| 112925 | glycoside hydrolase family 18 protein | Glycoside Hydrolases | 18 | 7.17595 | 9.86558 | 3.37407 |
| 500888 | Chitinase chi18-17 | Glycoside Hydrolases | 18 | 10.7537 | -0.26535 | -3.95398 |
| 148225 | endochitinase | Glycoside Hydrolases | 18 | 7.0282 | -0.26127 | -3.31599 |
| 479912 | glycoside hydrolase family 2 protein | Glycoside Hydrolases | 2 | 8.02007 | 7.24943 | 6.33391 |
| 524327 | N,O-diacetyl muramidase(GH25) | Glycoside Hydrolases | 25 | 10.0928 | 6.71248 | 3.93055 |
| 509041 | alpha-galactosidase | Glycoside Hydrolases | 27 | 5.2048 | 5.8812 | 1.92339 |
| 88799 | exo-rhamnogalacturonase b | Glycoside Hydrolases | 28 | 2.01235 | 5.6881 | 6.4049 |
| 502198 | beta-xylosidase | Glycoside Hydrolases | 3 | 2.88247 | 8.46206 | 7.57724 |
| 127782 | glycoside hydrolase family 3 protein | Glycoside Hydrolases | 3 | 1.09804 | -5.08827 | -0.29922 |
| 485240 | glycoside hydrolase family 30 protein | Glycoside Hydrolases | 30 | 5.2448 | 11.7243 | 7.33577 |
| 147762 | glycoside hydrolase family 30 protein | Glycoside Hydrolases | 30 | 6.12323 | 5.00051 | 4.42401 |
| 513145 | P2 protein (beta-1,6-glucanase) | Glycoside Hydrolases | 30 | 1.09636 | -8.21453 | -6.58603 |
| 149383 | glycoside hydrolase family 37 protein | Glycoside Hydrolases | 37 | 0.81564 | 4.84782 | 7.92076 |
| 512404 | hypothetical protein TRIATDRAFT_30270 | Glycoside Hydrolases | 5 | -3.52022 | 7.43661 | 6.08949 |
| 119344 | β-1,6-endoglucanase | Glycoside Hydrolases | 5 | 6.79576 | 5.04426 | 0.01647 |
| 513492 | fungal alpha-N-arabinofuranosidase | Glycoside Hydrolases | 54 | 6.03137 | 6.98384 | 4.02381 |
| 526018 | hypothetical protein | Glycoside Hydrolases | 55 | 3.21933 | 10.4047 | 9.66796 |
| 85422 | glycoside hydrolase family 72 protein | Glycoside Hydrolases | 72 | -0.8108 | 6.51911 | 2.79295 |
| 504171 | wd40 repeat-containing protein | Glycoside Hydrolases | 73 | 3.72514 | 8.02516 | 3.82927 |
| 515691 | glycoside hydrolase family 75 protein | Glycoside Hydrolases | 75 | 5.89253 | 9.31985 | 7.14687 |
| 45882 | Chitosanase (family 75) | Glycoside Hydrolases | 75 | 9.83244 | 6.11449 | 2.44109 |
| 11823 | glycoside hydrolase family 76 protein | Glycoside Hydrolases | 76 | 7.49582 | -4.31969 | -4.31969 |
| 96734 | glycoside hydrolase family 76 | Glycoside Hydrolases | 76 | 0.57033 | 9.93525 | 10.8109 |
| 479664 | glycoside hydrolase family 81 protein | Glycoside Hydrolases | 81 | 5.48351 | 6.26862 | 5.95991 |
| 273717 | alpha-l-fucosidase 2 | Glycoside Hydrolases | 95 | 4.31152 | 4.52384 | 5.83691 |
| 93968 | 1,2-α-L-fucosidases(GH95) | Glycoside Hydrolases | 95 | 5.80393 | 3.63545 | 2.82269 |
| 99474 | glycosyltransferase family 1 protein | GlycosilTransferases | 1 | -4.75103 | -2.98674 | -2.13483 |
| 10394 | polyketide synthase | GlycosilTransferases | 2 | -0.953623 | -2.46068 | -5.98207 |
| 510293 | conidial pigment polyketide synthase alb1 | GlycosilTransferases | 2 | -1.51372 | 6.25439 | 6.14396 |
| 504841 | conidiospore surface protein | GlycosilTransferases | 20 | 1.49367 | 6.64748 | 3.35946 |
| 9015 | capsule polysaccharide biosynthesis | GlycosilTransferases | 32 | 6.78003 | 0.709897 | -0.46347 |
| 125634 | trichothecene c-15 hydroxylase | GlycosilTransferases | 34 | 2.56185 | 6.27588 | 6.62881 |
| 521588 | isotrichodermin c-15 hydroxylase | GlycosilTransferases | 34 | 10.6739 | 6.96978 | -1.43531 |
| 509526 | cytochrome p450 | GlycosilTransferases | 34 | -0.6697 | -0.547131 | -5.1756 |
| 532775 | benzoate 4-monooxygenase cytochrome p450 | GlycosilTransferases | 34 | -0.01743 | -2.86095 | -6.51068 |
| 514166 | alpha beta hydrolase fold protein | GlycosilTransferases | 4 | -1.264 | -3.57995 | -6.96581 |
| 96797 | wsc domain-containing protein | GlycosilTransferases | 8 | 6.99977 | 11.4475 | 6.05176 |
| 619 | polysaccharide lyase family 7 protein | Polysaccharide Lyases | 7 | 7.84568 | -0.599013 | -1.89195 |
| 19572 | polysaccharide lyase family 8 protein | Polysaccharide Lyases | 8 | 7.92673 | 8.22775 | 5.88286 |
