# Supplementary material for: Identification of mycoparasitism-related genes against the phytopathogen Sclerotinia sclerotiorum through transcriptome and expression profile analysis in Trichoderma harzianum
Source: BMC Genomics. 2014 Mar 18;15:204. doi: 10.1186/1471-2164-15-204 (PMC4004048; doi:10.1186/1471-2164-15-204)
Supplement: Additional file 4: Table S2 — RNA-Seq sequencing and read mapping. [file 1471-2164-15-204-S4.pdf]

Additional file 2 - RNA-Seq sequencing and read mapping

Treatment	Total reads	[a]Filtered reads	%	[b]Mapped reads	%	[c]Unique reads	%	[d]Multi-reads	%	[e]Coverage
Glucose 12h	37899597	32557213	85,9	28022102	86.1	27795200	99.2	226902	0.8	109,0
Glucose 24h	37959229	32134980	84,7	28020488	87.2	27716697	98.9	303791	1.1	108,7
Glucose 36h	30650268	26414254	86,2	21435002	81.1	21292447	99.3	142555	0.7	83,5
SSCW 12h	30540926	26197356	85,8	22752680	86.9	22563628	99.2	189052	0.8	88,5
SSCW 24h	31695811	27310346	86,2	24904682	91.2	24720744	99.3	183938	0.7	97,0
SSCW 36h	30615037	26826959	87,6	16966713	63.2	16845349	99.3	121364	0.7	66,1

[a]Reads passing the quality check and the corresponding percentage (%) of total reads.

[b]Reads mapping to the *T. harzianum* CBS 226.95 genome version 1.0, and the corresponding percentage (%) of filtered reads.

[c]Reads mapping to unique locations in the *T. harzianum* genome, and the corresponding percentage (%) of mapped reads.

[d]Reads mapping to more than one location (≥ 2 matches) in *T. harzianum* genome, and the corresponding percentage (%) of mapped reads.

[e]Coverage of the *T. harzianum* CBS 226.95 genome version 1.0 (22.9 Mbp).
