# Supplementary material for: Identification of mycoparasitism-related genes against the phytopathogen Sclerotinia sclerotiorum through transcriptome and expression profile analysis in Trichoderma harzianum
Source: BMC Genomics. 2014 Mar 18;15:204. doi: 10.1186/1471-2164-15-204 (PMC4004048; doi:10.1186/1471-2164-15-204)
Supplement: Additional file 6: Table S7 — log2 Fold change expression of CAZy classes. The data shown is the average and standard deviation on each time condition. [file 1471-2164-15-204-S6.pdf]

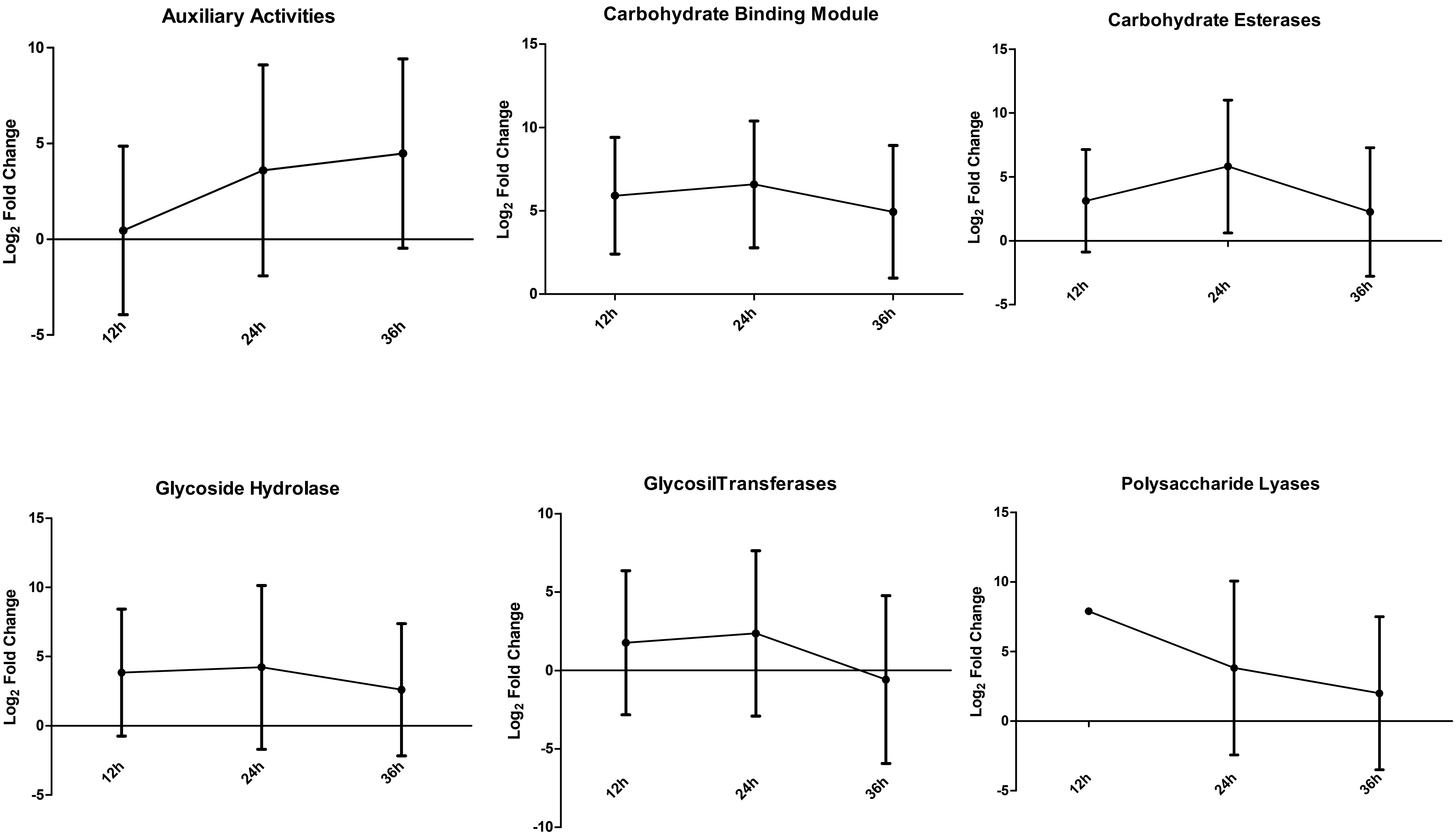

Additional file 7 - $\log_2$ Fold change expression of CAZy classes. The data shown is the average and standard deviation on each time condition.
