# Supplementary material for: Identification of mycoparasitism-related genes against the phytopathogen Sclerotinia sclerotiorum through transcriptome and expression profile analysis in Trichoderma harzianum
Source: BMC Genomics. 2014 Mar 18;15:204. doi: 10.1186/1471-2164-15-204 (PMC4004048; doi:10.1186/1471-2164-15-204)
Supplement: Additional file 7: Table S5 — Transporters differentially expressed in 12, 24 and 36 hours. [file 1471-2164-15-204-S7.docx]

Additional file 7: Table S5 – Transporters differentially expressed in 12, 24 and 36 hours

| **JGI ID** | **Putative Function** | **12h** | **24h** | **36h** |
| --- | --- | --- | --- | --- |
| 536643 | oligopeptide transporter | 8.65596 | -1.9913 | -2.86424 |
| 93925 | MFS transporter | 5.46782 | 4.01588 | 4.34789 |
| 151293 | MFS sugar transporter | 8.79979 | 6.35859 | 7.54647 |
| 487625 | hexose transporter | -4.7373 | -6.49868 | -5.30866 |
| 478895 | MFS peptide transporter ptr2 | 5.52247 | 1.59211 | -0.17848 |
| 546031 | MFS multidrug transporter | -0.87854 | -7.0279 | -0.24788 |
| 500863 | plasma membrane iron permease | 0.54794 | -4.80346 | -7.59663 |
| 525484 | Purine-cytosine permease | 0.32015 | -5.68397 | -9.22775 |
| 97417 | ABC transporter | -7.1545 | -6.72921 | -7.45898 |
| 319506 | MFS general substrate transporter | 6.75783 | 3.66801 | -1.76837 |
| 101937 | MFS oligopeptide transporter | 8.32326 | 7.59712 | 2.60933 |
| 17916 | hexose transporter | 7.62063 | 4.31425 | 1.2377 |
| 93830 | ammonium permease | -0.37122 | -1.4492 | -8.49687 |
| 76207 | MFS allantoate transporter | 4.5888 | 3.38088 | -5.16877 |
| 513160 | MFS multidrug transporter | -1.76456 | -2.87871 | -5.38088 |
| 127647 | mfs monocarboxylate transporter | -4.27852 | -3.56487 | -7.08955 |
| 142220 | MFS nitrate transporter | 0.21905 | 1.50734 | -11.3541 |
| 528008 | MFS multidrug resistant protein | -5.01216 | 1.58614 | -1.86288 |
| 112430 | efflux pump antibiotic resistance | -5.14409 | -4.57139 | -5.4247 |
| 529850 | oligopeptide transporter OPT | 7.89446 | 9.72302 | 9.09472 |
| 122877 | MFS transporter | -2.56045 | 10.1404 | 7.84387 |
| 478307 | MFS maltose permease | 6.38864 | 10.0851 | 7.6413 |
| 493261 | plasma membrane iron permease | 3.78604 | 7.97215 | 3.21457 |
| 506686 | general amino acid permease agp2 | 0.558702 | 5.6007 | 2.71614 |
| 15710 | ABC transporter | 5.29375 | 7.09588 | 5.59101 |
| 78602 | MFS carboxylic acid transport protein | -0.58224 | 10.0355 | 10.2595 |
| 78055 | MFS allantoate permease | 5.52008 | 6.00188 | 5.96806 |
| 11643 | alpha-glucosides permease mph2/3 | 4.63696 | 5.66278 | 5.85139 |
| 114756 | amino-acid permease inda1 | 1.77862 | 3.86686 | 6.20221 |
| 78790 | putative sugar transporter | 1.87711 | 4.145 | 4.86615 |
