# Supplementary material for: Identification of mycoparasitism-related genes against the phytopathogen Sclerotinia sclerotiorum through transcriptome and expression profile analysis in Trichoderma harzianum
Source: BMC Genomics. 2014 Mar 18;15:204. doi: 10.1186/1471-2164-15-204 (PMC4004048; doi:10.1186/1471-2164-15-204)
Supplement: Additional file 8: Table S6 — Bioanalyser profile of the six samples used to construct RNA-seq libraries. [file 1471-2164-15-204-S8.docx]

**Additional file 8: Table S6** – Bioanalyser profile of the six samples used to construct RNA-seq libraries
