# Supplementary material for: Identification of mycoparasitism-related genes against the phytopathogen Sclerotinia sclerotiorum through transcriptome and expression profile analysis in Trichoderma harzianum
Source: BMC Genomics. 2014 Mar 18;15:204. doi: 10.1186/1471-2164-15-204 (PMC4004048; doi:10.1186/1471-2164-15-204)
Supplement: Additional file 1: Table S8 — KEGG pathwayanalysisshownenzymesmappedin StarchandSucroseMetabolism. Arrowsin red, greenandblue (12, 24 and36h respectively) show theup-regulatedconditionofallgenes. [file 1471-2164-15-204-S1.pdf]

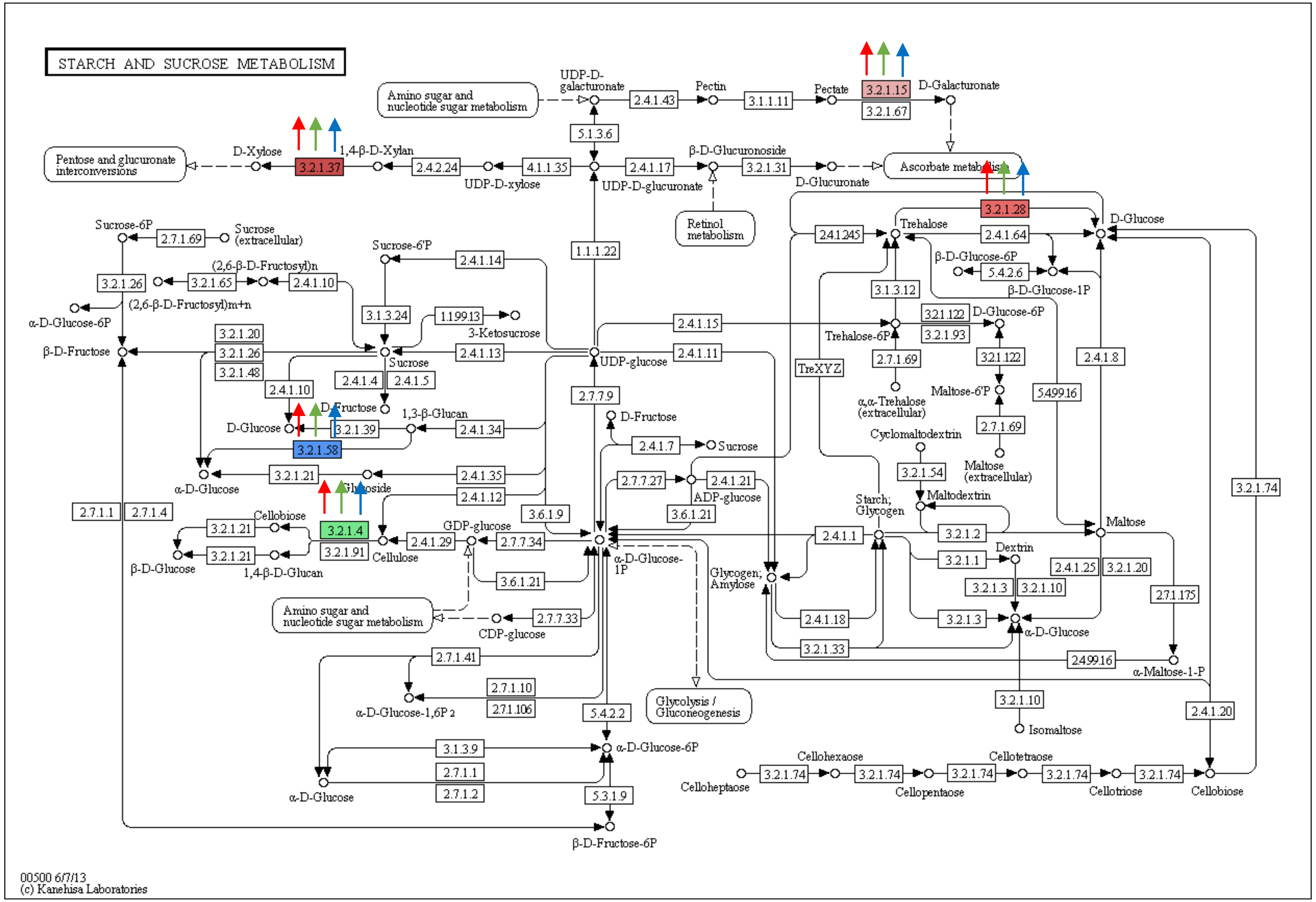

Additional file 9 – KEGG pathway analysis shown enzymes mapped in Starch and Sucrose Metabolism. Arrows in red, green and blue (12, 24 and 36h respectively) show the up-regulated condition of all genes.
